# Total and partial knee arthroplasty implants that maintain native load transfer in the tibia

**DOI:** 10.1302/2046-3758.112.BJR-2021-0304.R1

**Published:** 2022-02-16

**Authors:** Maxwell J. Munford, Jennifer C. Stoddart, Alexander D. Liddle, Justin P. Cobb, Jonathan R. T. Jeffers

**Affiliations:** 1 The Biomechanics Group, Department of Mechanical Engineering, Imperial College London, London, UK; 2 The MSk Lab, Department of Surgery and Cancer, Imperial College London, London, UK

**Keywords:** Bone strain, Porous implants, Additive manufacturing, tibial bone, total knee arthroplasty (TKA), bone-implant, titanium, unicompartmental knee arthroplasty (UKA), knee, tibial implants, proximal tibia, knee arthroplasty implants, cadaveric study

## Abstract

**Aims:**

Unicompartmental and total knee arthroplasty (UKA and TKA) are successful treatments for osteoarthritis, but the solid metal implants disrupt the natural distribution of stress and strain which can lead to bone loss over time. This generates problems if the implant needs to be revised. This study investigates whether titanium lattice UKA and TKA implants can maintain natural load transfer in the proximal tibia.

**Methods:**

In a cadaveric model, UKA and TKA procedures were performed on eight fresh-frozen knee specimens, using conventional (solid) and titanium lattice tibial implants. Stress at the bone-implant interfaces were measured and compared to the native knee.

**Results:**

Titanium lattice implants were able to restore the mechanical environment of the native tibia for both UKA and TKA designs. Maximum stress at the bone-implant interface ranged from 1.2 MPa to 3.3 MPa compared with 1.3 MPa to 2.7 MPa for the native tibia. The conventional solid UKA and TKA implants reduced the maximum stress in the bone by a factor of 10 and caused > 70% of bone surface area to be underloaded compared to the native tibia.

**Conclusion:**

Titanium lattice implants maintained the natural mechanical loading in the proximal tibia after UKA and TKA, but conventional solid implants did not. This is an exciting first step towards implants that maintain bone health, but such implants also have to meet fatigue and micromotion criteria to be clinically viable.

Cite this article: *Bone Joint Res* 2022;11(2):91–101.

## Article focus

- Can additively manufactured titanium lattice unicompartmental knee arthroplasty (UKA) and total knee arthroplasty (TKA) implants replicate the native load transfer in the proximal tibia?
- How do conventional solid titanium implants alter the load transfer in the proximal tibia?

## Key messages

- Titanium lattice UKA and TKA implants restored native loading in the tibia but conventional solid implants did not.
- By maintaining normal load transfer, this study provides an exciting and encouraging first step for the development of orthopaedic implants, which can maintain healthy bone for a longer portion of a patient’s lifetime.

## Strengths and limitations

- This study uses a manufacturing method already in widespread use in industry.
- The cadaver model closely replicates the mechanical properties of the patient’s bone.
- Only one loading condition was considered and the implants were not tested for fatigue strength and micromotion.

## Introduction

Knee arthroplasty procedures are highly successful interventions for the relief of pain and improvement in function of patients with osteoarthritis (OA); worldwide, 1.3 million procedures are performed annually.^1^ Conventional arthroplasty implants are made from solid metal, usually titanium or cobalt-chromium alloys. These alter the forces applied to the metaphyseal bone, which in turn disrupts the bone’s natural distribution of stress and strain, causing a detectable loss of bone quality as soon as two years post-surgery.^2,3^ This loss of bone quality and density in the tibia following arthroplasty is undesirable, but nevertheless tolerated because current-generation implants have excellent rates of survival, with typically 95% of cases recorded in joint registries surviving more than ten years.^4^ However, one in three patients under the age of 60 years will need a revision at some point in their life.^5^ An improvement to this status quo could be achieved if the periprosthetic bone density and strength were maintained throughout the life of the primary implant.

The problem of bone resorption is a consequence of the natural mechanical loading environment being disrupted by the presence of the implant.^6^ This can be due to loading, fixation method, or implant material and design. Furthermore, in cemented implant fixation, a reduction in bone volume of 85% at the bone-cement interface has been shown (six to ten years post-surgery).^7^ Similarly, variations in implant material and design choice have been shown to affect bone resorption.^8,9^ Bone formation and resorption are controlled by osteoblast and osteoclast cell activity, each stimulated by multiple factors including localized strain gradient.^10-12^ Models within the literature, such as Frost’s mechanostat, Wolff’s law, and Perren’s strain theory explain how this mechanism influences the complex and dynamic distribution of mechanical properties in bone.^10,13,14^ It may be possible to harness this remodelling process to maintain bone density after joint arthroplasty surgery. This would require implants with material properties that do not disturb the bone’s natural mechanical loading environment.^10,15^

Additive manufacture (AM) is a viable route to manufacture orthopaedic implants, and allows the creation of titanium lattices that control the strain experienced by bone and thus induce positive remodelling. Such lattices have been explored in a variety of materials, structures, and manufacturing methods to achieve control of pore size, anisotropy, and mechanical properties.^16-23^ Furthermore, combinations of such materials and structures have produced varying mechanical anisotropies.^24,25^ In animal models, these lattices can accelerate bone formation and increase bone density compared to solid metal controls.^18,21,26,27^ AM titanium structures have also been shown to offer improved long-term fixation which could prove beneficial in cementless arthroplasty.^28,29^ These findings indicate the huge potential of AM technology in orthopaedics, but have yet to be applied to an orthopaedic implant in a human model.

The hypothesis of our study was that unicompartmental knee arthroplasty (UKA) and total knee arthroplasty (TKA) tibial implants made from titanium lattice material could replace the tibial condyle surface, while minimizing disruption of the bone’s natural mechanical loading environment. A secondary aim was to explore whether a titanium lattice implant with a graded modulus throughout the implant would generate a more natural load transfer than one with a uniform modulus. This study was conducted in a human cadaveric model.

## Methods

### Implant design and manufacture

The study includes uncemented medial UKA and TKA tibial implants manufactured from conventional solid titanium and titanium lattice material. In all cases, a fixed-bearing polyethylene bearing surface was used. The TKA implant had a keel spanning the distance between each condyle’s dwell points.

The conventional solid UKA and TKA tibial implants were called M0 and T0 respectively. The titanium lattice UKA and TKA tibial implants were called M1 to M4 and T1 to T4, respectively (Table I). M1 and T1 had a uniform axial modulus of 0.6 GPa. M2 and T2 had an axial modulus of 0.6 GPa and transverse modulus of 0.45 GPa. M3 and T3 had a uniform axial modulus of 3.3 GPa. M4 and T4 had a graded axial modulus of 0.4 GPa to 0.7 GPa to match the stiffness gradient in the proximal tibia measured in a previous study.^30^

**Table I.** Description of implant variants tested; where mechanical properties of the proximal tibia have been matched, values were taken from existing literature.^26^ T0 to T4 refer to total knee arthroplasty designs. M0 to M4 refer to medial unicondylar knee arthroplasty designs.

Implant	Description	Mechanical modulus
T0, M0	Conventional solid implant	E_Axial_ = 113 GPa
T1, M1	Uniform axial modulus matched to the mean value in the proximal tibia	E_Axial_ = 0.6 GPa
T2, M2	Uniform axial and transverse modulus matched to the mean value in the proximal tibia	E_Axial_ = 0.6 GPa E_Transverse_ = 0.45 GPa
T3, M3	Uniform axial modulus set similar to that in the proximal tibia	E_Axial_ = 3.3 GPa
T4, M4	Graded axial modulus graded across the implant to replicate differences found across native condyles and subchondral depth, with a solid cortical rim	E_Axial_ = 0.4 to 0.7 GPa

Titanium lattice tibial components were made by filling the implant volume with a stochastic lattice structure.^24^ The diameter and connectivity of the lattice struts, and the strut density, were controlled to generate the desired stiffness. The relationship between these variables and stiffness were determined in previous work.^31^ This method uses Rhinoceros 6 and Grasshopper (Robert McNeel & Associates, USA) and is described in literature.^31^ The plateau surfaces and keels of all the lattice implants were manufactured from the stochastic lattice structure. There was no solid structure at all in these components.

All implants were manufactured using a Renishaw AM250 PBF additive manufacturing system (Renishaw, UK) with commercially pure titanium ASTM B348 grade 2 spherical powder (15 μm to 45 μm diameter), supplied by Carpenter Additive (USA). Laser power was constant at 50 W, while exposure times varied from 100 to 1,200 µs to achieve the desired apparent modulus. Specimens were heat treated at 750°C.

### Surgical planning and specimen preparation

Cadaveric human tissue was obtained from eight donors with no prior lower limb pathologies, traumas, or surgeries (age: 66 to 72 years; sex: eight male). Ethical permission was granted and investigations conformed with the research principles and study protocol as approved by the institution’s research ethics boards. Tissue was frozen within 24 hours post-mortem. Once thawed, all tissues apart from the extensor mechanism, collaterals, cruciates, capsule, and menisci were removed prior to surgical procedure.

**Fig. 1:**
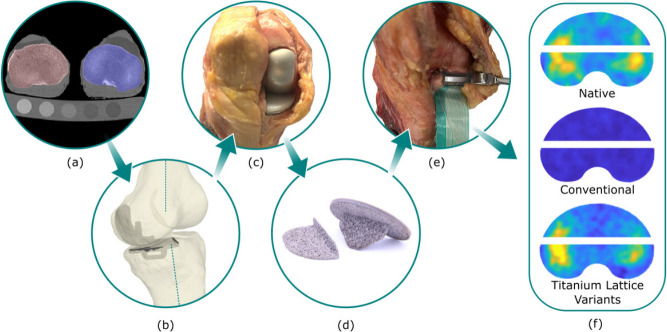
a) CT scan of specimens with calibration phantom. b) Surgical plans made based on specimen geometry and anatomical axes. c) Surgeries conducted and implants fit by a consultant surgeon. d) Additive manufactured unicompartmental knee arthroplasty (UKA) and total knee arthroplasty (TKA) tibial implants. e) Pressure film was placed at the bone-implant interface. f) Pressure maps were found for the native case, conventional implants, and titanium lattice implant variants.

A conventional CT scanner (SOMATOM Definition AS; SIEMENS AG, Germany) was used to image all specimens using a clinical imaging protocol (512 × 512 resolution, 120 kVp, 0.6 mm slice thickness, approximately 0.5 mm pixel spacing) with a five-material calibration phantom (Model 3; Mindways Software, USA) for bone mineral densitometry. Following scanning, 3D models of the specimens were segmented from the scan slices (Mimics, Materialise, Belgium), (Figure 1a).

Surgeries were planned on the basis of the preoperative CT to recreate mechanical alignment and constitutional varus (0° to 3°) (Figure 1b). Surgeries were conducted by a board-certified consultant orthopaedic knee reconstruction surgeon (ADL). First, a medial UKA was performed as planned using conventional instrumentation (Oxford Microplasty instruments, Zimmer Biomet, UK). Following the UKA tests, the surgeon performed TKA on the same specimens using specimen-specific cutting guides (Figure 1c). For all implanted cases, the tibial components used are listed in Table I, but on the femoral side conventional solid metallic femoral components were used in all cases.

### Knee loading

**Fig. 2:**
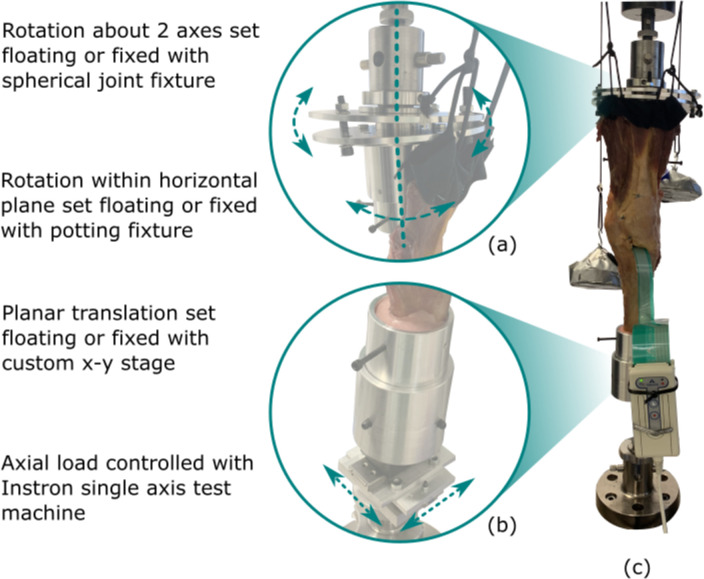
a) Native alignment and condylar dwell points were found with custom fixtures to set rotational degrees of freedom and b) translational degrees of freedom floating or fixed before c) testing specimens in extension under body weight axial loading.

The proximal femur and distal tibia were potted in fixtures aligned to their anatomical axes using intramedullary rods to align in the sagittal and coronal planes. To ensure a natural alignment for each specimen, there were three rotational degrees of freedom on the femoral fixture (Figure 2a), two translational degrees of freedom (anteroposterior (AP) and mediolateral (ML)) on the tibial fixture (Figure 2b), and the third translational degree of freedom (axial) was the direction of the actuator of the materials testing machine (Instron 8872, 10 kN load cell). All degrees of freedom were initially released to allow the femur and tibia to align according to the loads applied, such that small angular deviations could occur due to the deflection of the components and underlying bone. Specimens were placed in 0° flexion and an axial load of 700 N was applied to find the natural position of the tibia relative to the femur. This moved the femur into 5° to 7° varus relative to the tibia. For each specimen, once this position was found, all degrees of freedom were fixed to ensure the same loading was applied to that specimen for native, UKA, and TKA cases. Loading was applied in position control at 2 mm/min to a magnitude of 700 N, where it was then held in load control, and contact pressure data captured immediately to minimize any viscoelastic effects. The 700 N load is representative of the contact force during two-legged stance (Figure 2c). In addition, the extensor mechanism was tensioned at 21 N to provide an upward force from the tibial tubercle.^32^

Contact stress at the tibial bone-implant interface was measured with pressure film sensors (Tekscan, USA) following a similar method to Verstraete et al.^33^ The UKA tibial bone cuts were made, bone fragment kept in place, a single pressure sensor (I-scan 4011) placed in the transverse cut, and contact pressure captured under the applied load. The femoral bone cuts were then made and the femoral component inserted. Each of the five UKA tibial components (M0 to M4) were subsequently implanted in randomized order and contact pressures again captured under the same applied load. The TKA tibial bone cuts were then made and the process repeated for the TKA implants except two sensors (I-scan 5011) were used anterior and posterior to the tibial keel respectively (Figures 1d to 1f). In the native case for both UKA and TKA, a transverse cut was necessary to accommodate a pressure sensor. It would not have been possible to gather the native case pressure data without making this cut, but clearly making the cut could influence the load transfer. To mitigate this limitation, we performed a finite element analysis of the intact tibia versus one with the transverse cut made, and contact pressures were within 3% for all cases. For this reason, we deemed that the transverse cut in the native case was acceptable.

### Statistical analysis

A histogram plot of the pressure sensor data was captured to allow comparison between the natural, UKA, and TKA cases. Similarity of the bone-implant interface stress distribution between each implant and the native case was measured as the Jaccard similarity (S), which is defined as the intersection of stress surfaces divided by the union of stress surfaces.^34^ The percentage of interface area underloaded (A) relative to native bone was calculated directly from the pressure sensor histogram data. One-way analysis of variance (ANOVA) with Bonferroni post-hoc tests was performed on the Jaccard similarity and maximum stress in each condyle, with implant variant as a factor group. For TKA maximum stress data, both condyles were considered separately with independent-samples *t*-tests performed to determine any differences between them.

## Results

### UKA results: stress distribution

**Fig. 3:**
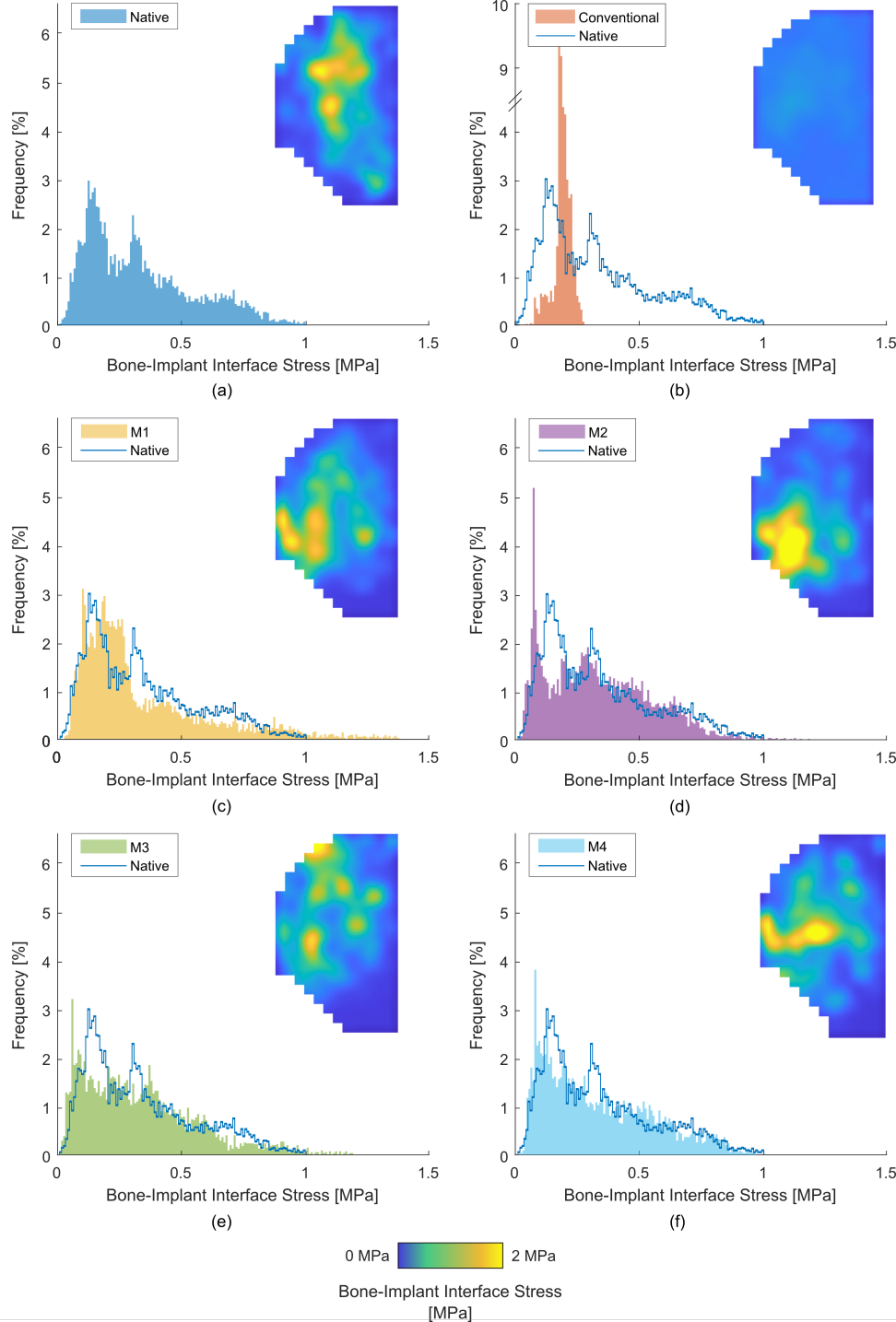
Average (across eight specimens) stress maps and histogram plots of bone-implant interface stress in unicompartmental knee arthroplasty for: a) the native case; b) the conventional implant (M0); and c) to f) the additive manufactured lattice variants: c) M1, d) M2, e) M3, and f) M4. Note the break in axis of Figure 3b.

The titanium lattice implants (M1 to M4) replicated the native stress distribution in the medial condyle (Figure 3). Stress at the bone-implant interface was at least 71.2% similar to the native case, with less than 5% of interface area underloaded (Table II). For the conventional implant (M0), stress distribution was only 19.8% similar to the native case. This resulted in bone immediately beneath the femoral condyle contact points being comparatively underloaded. This underloaded bone covered 71% of the bone-implant interface area.

**Table II.** Mean measures for similarity of stress distribution (%), percentage of interface area underloaded compared to the native case (%), maximum stress (MPa) in medial unicompartmental knee arthroplasty, and the total load measured at the medial bone-implant interface (N). M0 refers to the conventional implant; M1 to M4 refers to the additively manufactured implants as given in Table I.

Test variant	Mean Jaccard similarity, % (SD)	Mean area underloaded, % (SD)	Mean maximum stress, MPa (SD)	Mean total load measured, N (SD)
Native	N/A	N/A	2.0 (0.7)	422.9 (1.1)
M0	19.8 (0.6)	71.1 (1.2)	0.2 (0.0)	386.8 (1.3)
M1	76.9 (1.8)	1.2 (1.0)	1.7 (0.6)	362.6 (1.8)
M2	74.3 (1.1)	3.6 (2.1)	1.7 (0.8)	374.4 (2.2)
M3	71.2 (2.3)	5.0 (1.7)	2.4 (0.9)	423.5 (2.6)
M4	75.4 (3.0)	2.7 (2.2)	1.8 (0.8)	420.0 (2.3)

N/A, not applicable; SD, standard deviation.

### UKA results: maximum induced stress

**Fig. 4:**
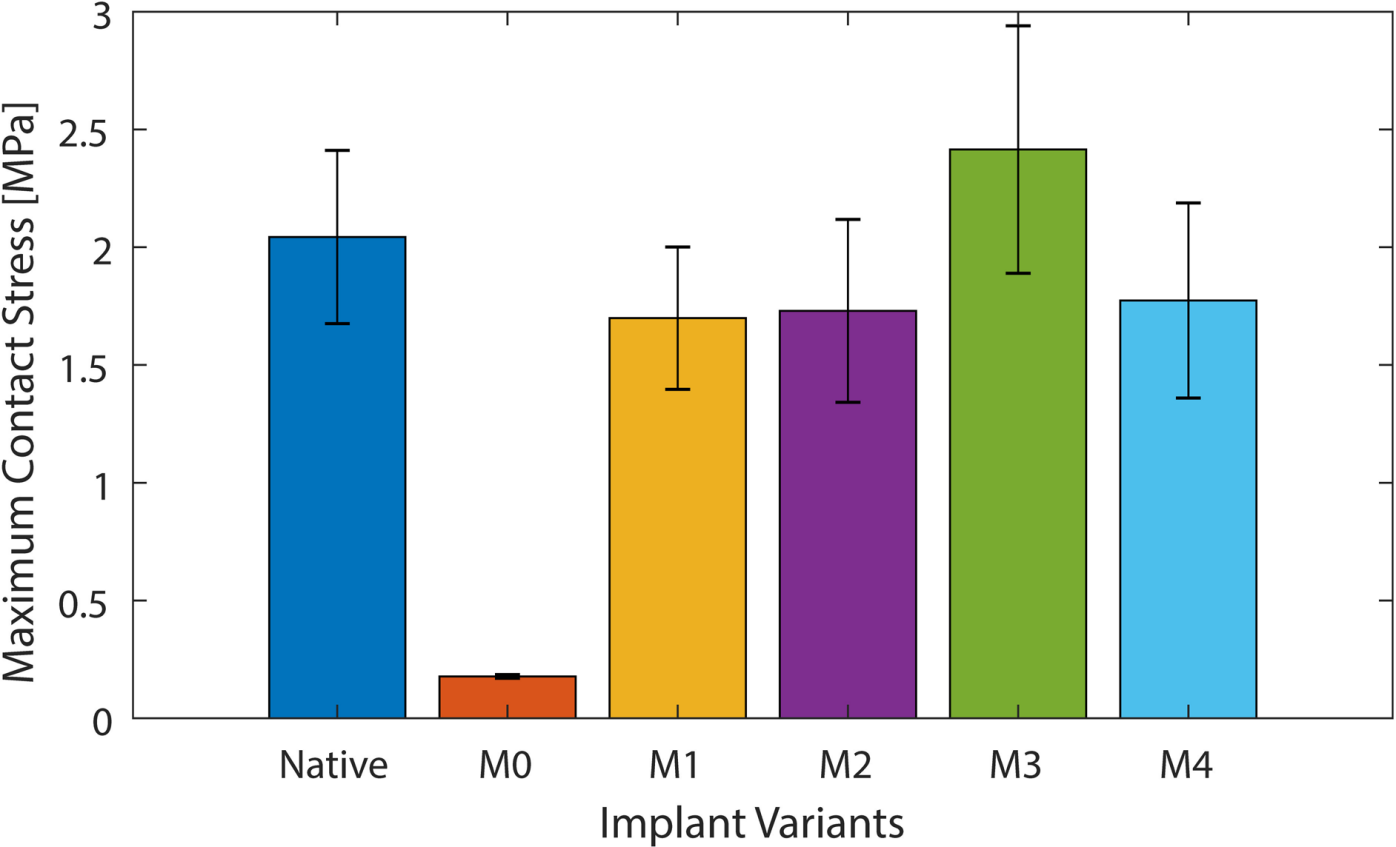
Maximum stress for medial unicompartmental knee arthroplasty loading, for the native knee, conventional implant (M0), and the four additive manufactured variants (M1, M2, M3, and M4).

The maximum stress in the medial condyle was not changed by the titanium lattice implants (M1 to M4) compared to the native case (p = 0.991, 0.993, 0.998, 0.995 for M1 to M4 respectively) and was between 1.5 MPa and 2.5 MPa for all cases (Figure 4, Table II). No difference was detected between M1 and M4. For the conventional implant (M0), the maximum stress was ten-times lower than the native case (p = 0.023) with a value of 0.2 MPa (Figure 4, Table II). The position of the peak stress after arthroplasty was variable in the anterior-posterior direction; this may have been due to the resection of the medial meniscus.

### TKA results: stress distribution

**Fig. 5:**
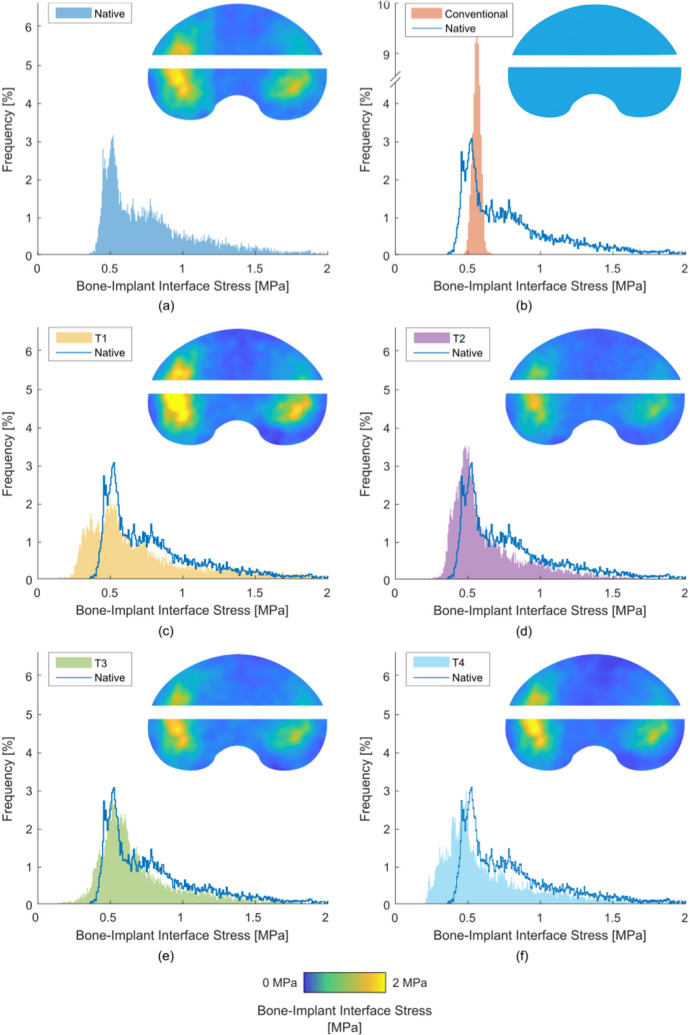
Average (across eight specimens) stress maps and histogram plots of bone-implant interface stress in total knee arthroplasty for: a) the native case; b) the conventional implant (T0); and c) to f) the additive manufactured lattice variants: c) T1, d) T2, e) T3, and f) T4. Note the break in axis of Figure 5b.

Across the whole bone-implant interface, the titanium lattice implants (T1 to T4) replicated the native stress distribution in the medial condyle but there was no difference between M1, M2, M3, or M4 (Figure 5). Stress at the bone-implant interface was at least 67.8% similar to the native case, with less than 1.7% of interface area underloaded (Table III). For the conventional implant (T0), stress distribution was only 20.5% similar to the native case. This resulted in bone beneath the dwell points being comparatively underloaded. This underloaded bone covered 77% of the bone-implant interface area.

**Table III.** Mean measures for similarity of stress distribution (%), percentage of interface area underloaded compared to the native case (%), maximum stress (MPa) in total knee arthroplasty, and the total load measured across the whole bone-implant interface (N). T0 refers to the conventional implant; T1 to T4 refers to the additively manufactured implants as given in Table I.

Test variant	Mean Jaccard similarity, % (SD)	Mean area underloaded, % (SD)	Mean maximum stress, MPa (SD)		Mean total load measured, N (SD)
			Medial condyle	Lateral condyle	
Native	N/A	N/A	2.1 (0.1)	1.7 (0.2)	698.4 (1.4)
T0	20.5 (2.4)	77.0 (1.2)	0.2 (0.0)	0.2 (0.0)	698.8 (0.8)
T1	77.6 (5.0)	0.9 (0.8)	2.7 (0.7)	1.9 (0.2)	699.1 (0.6)
T2	67.8 (12.1)	0.5 (0.4)	1.8 (0.3)	1.4 (0.2)	699.2 (0.2)
T3	74.2 (5.8)	1.7 (0.5)	2.2 (0.1)	1.7 (0.3)	697.8 (1.3)
T4	72.9 (8.1)	0.5 (0.3)	2.0 (0.2)	1.4 (0.2)	698.5 (0.7)

N/A, not applicable; SD, standard deviation.

### TKA results: maximum induced stress

In the medial condyle, the maximum stress was not changed by the presence of three out of four of the titanium lattice implants (T2 to T4) compared to the native case (p = 0.891, 0.910, 0.978 for T2 to T4 respectively) and was between 2 MPa and 2.5 MPa in these cases. The T1 titanium lattice implant generated a maximum stress 1.3 times greater than the native case (p = 0.027) with a value of 2.7 MPa. The conventional implant (T0) generated a maximum stress 10.5-times lower than the native case (p = 0.019) with a value of 0.2 MPa.

In the lateral condyle, the maximum stress was not changed by the presence of the titanium lattice implants (T1 to T4) compared to the native case (p = 0.989, 0.723, 0.961, 0.795 for T1 to T4 respectively) and was between 1.5 MPa and 2 MPa in all cases. The conventional implant (T0) generated a maximum stress 8.5-times lower than the native case (p = 0.027) with a value of 0.2 MPa.

**Fig. 6:**
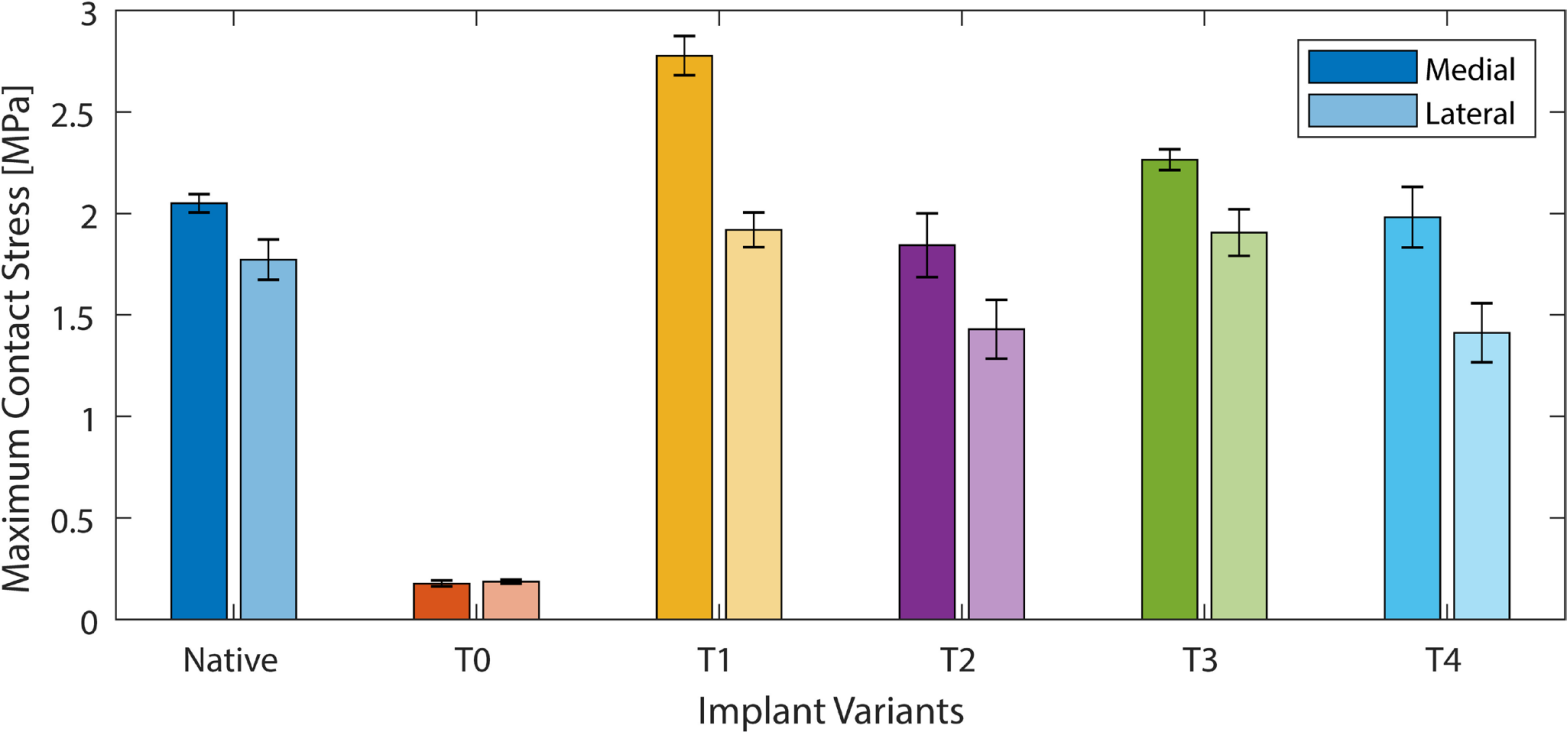
Maximum stress for each condyle in total knee arthroplasty loading, for the native knee, conventional implant (T0), and the four additive manufactured variants (T1, T2, T3, and T4).

In the native case, the maximum stress in the medial condyle was 20% greater than that in the lateral side (p = 0.031). For the titanium lattice implants (T1 to T4), the maximum stress in the medial condyle was 30% to 40% greater than in the lateral side (Figure 6, Table III) (p = 0.013, 0.021, 0.018, 0.009 for T1 to T4 respectively). For the conventional implant (T0), no difference was found between stresses in the medial and lateral condyle (p = 0.998).

## Discussion

The most important finding of this study is that titanium lattice UKA and TKA implants can provide a load transfer in the proximal tibia that is very close to the native un-implanted bone, while conventional solid titanium implants underloaded 71% and 77% of the tibial bone surface area, respectively. Both UKA and TKA titanium lattice implants generated the same magnitude and distribution of stress across the tibia as native bone, while conventional UKA and TKA implants generated peak stress magnitudes in the bone 10- and 9.7-times lower than the native case respectively. By maintaining normal load transfer to the bone, the titanium lattice implants may provide the mechanical conditions for normal bone remodelling throughout the implant’s life.

Our data can be compared to clinical observations of cementless trabecular metal-type tibial implants that have lower stiffness than conventional implants. A 13-year randomized controlled trial of such a device found better patient outcomes and radiological findings compared to a conventional metal-backed design.^35^ This improved performance may have been partially due to the more normal load transfer for the trabecular metal design. Our data can also be compared to computational models of load transfer in the tibia.^33,36-38^ These models report stresses in the intact native tibia that are centred around contact focal points, and range from 0.06 MPa to 2.4 MPa.^37,39,40^ They also predict bone-implant interface stress to be 0.03 MPa to 0.15 MPa after conventional medial UKA and TKA.^37,38,40^ The same computational analyses reported that reduced modulus implants (0.7 GPa to 1.5 GPa) engender a bone-implant interface stress of 0.9 GPa to 1.8 GPa.^39,41^ Our data concur with all these prior findings, but our study is the first to manufacture such implants and demonstrate this in a human cadaveric study.

In addition to measuring load transfer, we had an unexpected finding related to the loadshare between the medial and lateral condyle. Many sources in literature observe that most of the load in the native knee is transmitted through the medial condyle.^33^ For the native knee, our data matched this, with 53% to 62% of the load beneath the condyle concentrated on the medial side. However, when the conventional TKA was performed, the medial load share dropped to 48% to 52%. So while the adductor moment defines the medial/lateral load share at the bearing surface of the knee, the stiff tibial base plate of the conventional TKA may cause a more uniform load transfer to the bone with reduced medial bias.^42^ This has been observed previously.^39^ Conversely, the compliance of the titanium lattice implants may have allowed the resultant force to be more on the medial side, similar to the native case.

A limitation of this study is that we used cadaveric specimens, and assume the data translate to living bone. To minimize this, we used fresh-frozen specimens, not embalmed, and left all tissues that were critical to the loading intact. Similarly, the sample size of specimens used was relatively small and contained no female donors, however the power, significance, and effect sizes of statistical analysis used were deemed acceptable. We also did not screen our specimens to have knee OA, and the bone properties of our specimens may not be representative of patients, particularly those with medial OA where the medial bone may have remodelled to be different from normal. Another limitation was that the loading situation was a simplified example of someone standing still (full extension at body weight loading), whereas in reality a spectrum of loading conditions are applied to the proximal tibia. The total load seen at the bone-implant interface was consistent between all UKA and TKA tests respectively (Table II and Table III). This study measured only contact stress at the bone-implant interface rather than internal stress and strain distribution within the bone. We were also unable to measure pressure at the keel, but an ingrown keel, particularly in TKA, could transmit high forces. Further work is needed to explore the effects of additive manufactured implants on the keel and internal strains in the bone – computational methods could be particularly suited to this. A technical limitation was that the method of measuring contact stress in the native case required a transverse slot to be cut in the bone to place the sensor at the site of the bone-implant interface. The effects of this limitation were deemed to be acceptable following a finite element analysis, which measured differences in contact stress between our experimental case and intact tibia of less than 3%. This was modelled with loading of 700 N over a 60:40 medial:lateral load split and boundary conditions for the proximal tibial piece(s) applied from literature.^43,44^ A final limitation is that the lattice implant variants 1 to 4 represent a purist approach of matching the bone properties to demonstrate what is possible – future development of this concept would need design compromises to meet fatigue loading requirements, such as ISO 14879-1:2020. However, the additive manufacturing method is ideally suited to reach a compromise between load-sharing and fatigue strength, because solid reinforcing elements can be built into the design at the computer-aided design stage.

The clinical relevance of this work is that titanium lattice implants can restore native loading in the human knee following UKA and TKA, which could improve the maintenance of bone density following UKA and TKA procedures. This is important because loosening causes 30% and 17% of implant failures, in UKA and TKA respectively, and peri-prosthetic bone resorption often presents a significant problem in the revision procedure.

In conclusion, this study showed that normal load transfer in the proximal tibia can be maintained after knee arthroplasty (UKA and TKA) by using additive manufactured titanium lattice tibial components. By maintaining normal load transfer, this study provides an exciting and encouraging first step for the development of orthopaedic implants which can maintain healthy bone for a longer portion of a patient’s lifetime.
